# Structure of CfaA Suggests a New Family of Chaperones Essential for Assembly of Class 5 Fimbriae

**DOI:** 10.1371/journal.ppat.1004316

**Published:** 2014-08-14

**Authors:** Rui Bao, April Fordyce, Yu-Xing Chen, Annette McVeigh, Stephen J. Savarino, Di Xia

**Affiliations:** 1 Laboratory of Cell Biology, Center for Cancer Research, National Cancer Institute, National Institutes of Health, Bethesda, Maryland, United States of America; 2 Enteric Diseases Department, Infectious Diseases Directorate, Naval Medical Research Center, Silver Spring, Maryland, United States of America; 3 Hefei National Laboratory for Physical Sciences at Microscale and School of Life Sciences, University of Science and Technology of China, Hefei, Anhui, People's Republic of China; 4 Department of Pediatrics, Uniformed Services University of the Health Sciences, Bethesda, Maryland, United States of America; University of California, Davis, United States of America

## Abstract

Adhesive pili on the surface of pathogenic bacteria comprise polymerized pilin subunits and are essential for initiation of infections. Pili assembled by the chaperone-usher pathway (CUP) require periplasmic chaperones that assist subunit folding, maintain their stability, and escort them to the site of bioassembly. Until now, CUP chaperones have been classified into two families, FGS and FGL, based on the short and long length of the subunit-interacting loops between its F1 and G1 β-strands, respectively. CfaA is the chaperone for assembly of colonization factor antigen I (CFA/I) pili of enterotoxigenic *E. coli* (ETEC), a cause of diarrhea in travelers and young children. Here, the crystal structure of CfaA along with sequence analyses reveals some unique structural and functional features, leading us to propose a separate family for CfaA and closely related chaperones. Phenotypic changes resulting from mutations in regions unique to this chaperone family provide insight into their function, consistent with involvement of these regions in interactions with cognate subunits and usher proteins during pilus assembly.

## Author Summary

Bacterial infection begins with microbial adhesion to host cells. For gram-negative bacteria, adhesion is often mediated by pili, proteinaceous polymers that protrude from the bacterial surface and recognize host receptors. During assembly, each pilus protein subunit is assisted in folding by a chaperone that shuttles the subunit to an outer membrane usher complex, which serves as assembly platform. There, the chaperone transfers its subunit cargo into the growing pilus polymer, which protrudes out the usher pore. Here, we present the crystal structure of CfaA, the chaperone protein of the CFA/I pilus. The CFA/I pilus is the archetypal colonization factor (CF) for enterotoxigenic *Escherichia coli*, a major cause of life-threatening, dehydrating diarrhea in young children of low-income countries and in travelers to these regions. This structure reveals unique features that allow us to define a new class of chaperones that assist pilus assembly in bacteria. Probing these unique features with site-direct mutagenesis, we were able to gain new insight into the mechanism of pilus assembly.

## Introduction

Bacteria assemble filamentous projections on their surface to facilitate adhesion to other bacteria, eukaryotic cells and abiotic substrates. These macromolecular organelles are composed of protein polymers and can appear as regular, rod-like pili (or fimbriae), irregular, thin fibrils or indistinct structures. In gram-negative bacteria, many of these organelles are assembled by the chaperone-usher pathway (CUP). The three essential components of this pathway are one or more pilin subunits capable of polymerization, a periplasmic chaperone that catalyzes proper folding of the pilin subunits and shuttles them to the outer membrane for assembly, and an outer membrane usher that orchestrates ordered tip-to-base polymerization [1].

Extensive work on P pili and type 1 fimbriae from uropathogenic *E. coli* (UPEC) and related CUP fimbriae has yielded well-founded models of pilus bioassembly by the CUP [1]. Crystal structures of their evolutionarily related periplasmic chaperones, PapD and FimC, respectively, reveal two immunoglobulin (Ig)-like domains arranged in a boomerang shape [2]–[4]. Upon export of a nascent pilin subunit into the periplasm, a β-strand in the N-terminal domain of the chaperone fills a hydrophobic cleft in the pilin to provide the missing G strand in an otherwise incomplete Ig-like pilin subunit, a mechanism called donor-strand complementation (DSC) [3], [4]. The chaperone-pilin complex docks with the outer membrane usher and inserts a supernumerary N-terminal pilin β-strand into the hydrophobic groove of a foregoing pilin, thereby displacing the chaperone G1 strand from the latter by a ‘zip-in, zip-out’ process called donor strand exchange (DSE) [5]. Ordered iterations of this cycle drive pilus elongation and extrusion from the bacterial surface through the usher pore.

All chaperones of the CUP share certain structural motifs and highly conserved residues that are vital to its chaperone and transfer functions [6], [7]. This chaperone superfamily has been differentiated by sequence analysis into two subgroups, in which the loop between the F1 and G1 strand of the chaperone is either short (FGS) or long (FGL) [6]. The FGS family chaperones have a characteristic short subunit-interacting loop (on average 13 residues) between β-strands G1 and F1 in the central conserved β-sheet and are confined exclusively to the bioassembly of rod-like pili such as P pili or type 1 pili. By contrast, those in the FGL family feature a long interacting loop (on average 24 residues) between β-strands G1 and F1 and take part only in the assembly of atypical filaments, such as the F1 antigen of *Yersinia pestis*. The exact nature of chaperone-pilin interaction differs for these two groups, although both conform to the common mechanism of donor-strand complementation and exchange.

Another family of regular rod-like bacterial filaments, designated as Class 5 fimbriae [8] includes eight members that are produced by enterotoxigenic *Escherichia coli* (ETEC), a predominant cause of dehydrating diarrhea in travelers and young children in low-income countries. In studies of one such ETEC fimbria, CS1, the essential role of each of the four proteins encoded by the CS1 operon was experimentally defined [9]–[13]. These are a minor subunit (CooD) required for initiation of fimbrial assembly and adhesion, a major subunit (CooB) that is the primary antigenic determinant, a periplasmic chaperone (CooA) that stabilizes nascent structural subunits, and an outer membrane protein (CooC) presumed to serve an usher-like function. Thus, the CS1 bioassembly components have functional counterparts in the CUP, but none share any primary sequence similarity. This prompted speculation that Class 5 fimbriae evolved along a convergent evolutionary path [10] and evoked its designation as the ‘alternate’ chaperone pathway (ACP) [14]. Although none of the chaperones for Class 5 fimbriae were included in the study that led to the classification of FGL and FGS chaperones [6], the rod-like morphology of Class 5 fimbriae has presumptively suggested the association of all chaperones in this class with the family of FGS chaperones [15].

In more recent studies of CFA/I fimbriae, the archetypal Class 5 ETEC fimbria, the crystal structures of its minor (CfaE) and major (CfaB) structural components were solved [16], [17]. The lack of primary sequence similarity notwithstanding, each of these subunits generally conforms to the Ig-like domain structure of corresponding subunits of P pili and type 1 fimbriae. These and other studies clearly implicate the mechanism of donor-strand complementation and exchange in bioassembly of CFA/I fimbriae, suggesting that Class 5 fimbriae may actually have diverged from CUP fimbriae in the very distant past [18]. This view is substantiated in a more recent phylogenetic analysis of fimbrial usher protein sequences, which classified all Class 5 pili into a separate group or α clade that diverged from other CUP clades [19]. Interestingly, while this usher-based phylogeny categorizes all pili with FGL chaperones into a single clade (γ3 clade), those with FGS chaperones were grouped into several distinct clades (β-, γ1-, γ2-, γ4-, κ-, and π-fimbriae) that are not more closely related to each other than to the FGL systems, calling into question whether chaperones of rod-shaped Class 5 pili should all be placed into the FGS family.

In this report, we present the crystal structure of the CFA/I pilus chaperone protein CfaA. Structure-based sequence alignment indicates that chaperone proteins of Class 5 pili constitute a family that is distinct from the FGS and FGL families. Mutations in sequence motifs that are unique to the Class 5 chaperones result in measurable functional changes of CfaA consistent with our hypothesis and further suggest that the unique features in Class 5 pilus chaperones dictate their interactions with cognate subunits and usher proteins.

## Results

### Overview of CfaA structure

The full-length CfaA chaperone (residues 1–218) was expressed with a C-terminal hexahistidine tag and recovered from the periplasmic fraction post-cleavage of its 19-residue signal peptide. Mature CfaA was purified to homogeneity and crystallized. CfaA crystals diffracted X-rays well, revealing the symmetry of space group *C*2. Initial crystallographic phases were obtained experimentally by the method of multiple isomorphous replacement coupled with anomalous scattering (MIR/AS) using platinum and lead derivative data sets with an overall figure of merit of 0.48 (Table 1). The final atomic models were refined using either native or derivative data sets with the best resolution to 1.8 Å.

**Table 1:** Statistics on qualities of diffraction data sets, phasing and refined models.

***Data and MIR/AS phasing***			
Data Set	Native^a^	Pb	Pt
Wavelength (Å)	1.54178	0.89197	0.98197
Resolution (Å)	2.03	1.77	2.80
No. unique reflections	14,999	22,616	5,596
R_merge_	0.066	0.053	0.084
Completeness	97.4	99.3	95.1
Mean figure of merit for MIR/AS	0.48		
***Model refinement***			
R_work_ (%)	21.0 (36.3)	20.4 (27.0)	19.8 (22.9)
R_free_ (%)^b^	25.5 (38.5)	24.7 (29.1)	25.8 (30.3)
No. of residues	1,687	1,659	1,677
No. of non protein atoms	122	130	32
Mean B factor (Å^2^)	45.0	40.7	36.9
Rmsd for bond lengths (Å)	0.013	0.01	0.018
Rmsd for bond angles (°)	1.5	1.2	1.6
Ramachandran plot (%)			
Favored	96.6	95.6	95.0
Allowed	3.4	4.4	5.0
Disallowed	0	0	0

a Both native and derivatized crystals have the symmetry of space group C2.

b 10% of total reflections were set aside for the R_free_ calculation.

**Figure 1:**
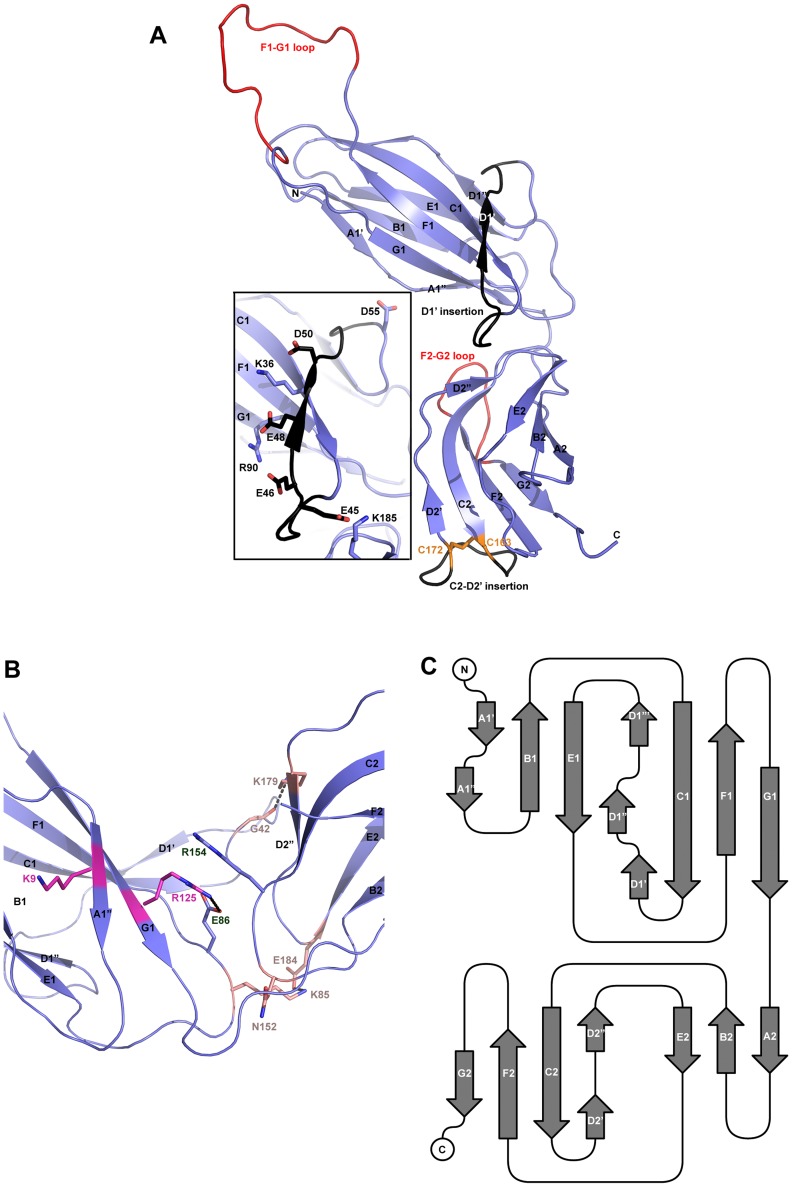
Structure of CfaA chaperone of Class 5 fimbriae. (A) Ribbon representation of the structure of monomeric CfaA. The seven strands in the N-terminal domain are labeled sequentially from A1–G1, and similarly in the C-terminal domain as A2–G2. Those segments in the G1–F1 and G2–F2 loops, which are crystallographically disordered, are shown in red. Insertions such as the D1′ and C2–D2′ insertions that are unique to Class 5 family chaperones are colored black and labeled. The disulfide bridge stabilizing the C2–D2′ insertion is shown as stick model in orange and labeled. Inset: detailed charge interactions in the acidic D1′ insertion is displayed. Five acidic residues in the insertion are shown as stick model (carbon atoms in black and oxygen in red), as are the interacting basic residues (with carbon in light blue and nitrogen in dark blue). (B) Interaction in the cleft formed at the interface between the N- and C-terminal domains of CfaA, showing interactions by charged residues in stick models with nitrogen in blue and oxygen in red. Residues K9 and R125 are shown with carbon in light magenta. Residues E86 and R154 are shown with carbon in light blue. Other interacting residues are shown with carbon in orange. (C) Schematic diagram depicting the topological arrangement of β-strands in CfaA structure.

As with all CUP chaperone structures previously described [2], [20]–[24], the overall structure of CfaA adopts a boomerang shape. The N- (1–129) and C-terminal (130–218) domains form two lobes angled at 121 degrees, as measured along the longest inertial vectors for the two domains, with a deep interlobe cleft (Fig. 1A). Based on structural comparison of other chaperones in the presence and absence of bound subunit, this spatial arrangement of the two lobes is reportedly rigid [3], [5], [25], [26]. We predict that such structural rigidity is preserved in the CfaA structure due to the extensive interactions that exist between the two domains, including either water-mediated or direct hydrogen bonding interactions and van der Waals contacts represented by a buried interdomain surface area of 1191 Å^2^ (Fig. 1B and Tables S1 and S2).

Each domain is represented by a seven-stranded β-barrel with a typical immunoglobulin (Ig) fold (Figs. 1A and 1C). Despite an overall low average temperature factor (B factor) of 40.8 Å^2^, the N-terminal domain displays a significantly lower average B factor (26.8 Å^2^) than the C-terminal domain (59.9 Å^2^). This is due to the self-dimerization or self-capping of the N-terminal domain with the same domain of a neighboring molecule in the crystal (see below). Discontinuous electron densities were observed for residues 98–114 of the loop between the F1 and G1 strands of the N-terminal domain and for the loop (residue 203–209) between the F2 and G2 strands of the C-terminal domain, which were similarly observed in isolated PapD and SafB chaperone structures [5], [7].

In the absence of bound pilins, chaperone proteins have been shown to dimerize in order to protect their interactive surface from nonspecific aggregation. This has been called self-capping oligomerization in PapD and Caf1M chaperones [7], [23]. Although there is one CfaA molecule present in a crystallographic asymmetric unit, application of the crystallographic two-fold symmetry generates a dimer that is self-capped by two adjoining G1 strands, presenting a continuous β-sheet between the two subunits (Fig. S1).

### A novel family of chaperones revealed by structure-based sequence alignment

**Figure 2:**
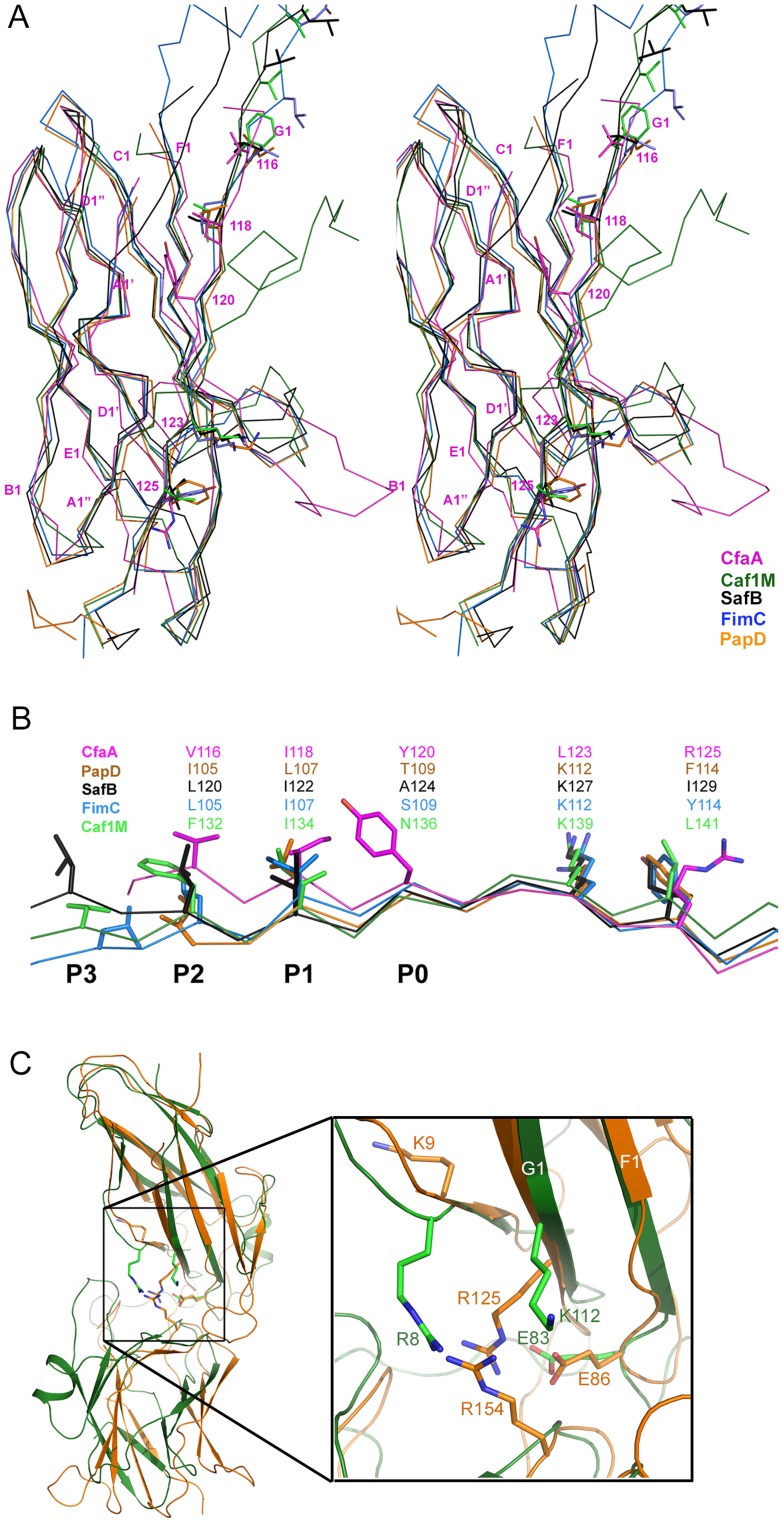
Structure comparison of chaperones from different families. (A) Stereoscopic pair showing the superposition of structures of chaperone N-terminal domains. Five structures are included: CfaA (magenta), Caf1M (green), SafB (black), FimC (blue) and PapD (coral). All β-strands are labeled. Subunit interacting residues on the G1 strand are shown as stick models and numbered. (B) Detailed alignment of the donor strand for CfaA, Caf1M, SafB, FimC and PapD, showing alternating hydrophobic residues at positions P0 (hydrophobic, CfaA only) and P1 to P3. (C) Structure comparison of CfaA and PapD. Structures of CfaA (coral) and PapD (green) are superimposed and the cleft region between the two domains is magnified. Charged residues K9, E86, R125, and R154 from CfaA are shown as stick models with carbon atoms color in coral. Those from PapD R8, E83, and K112 are similarly illustrated with carbon in green. Oxygen atoms are in red and nitrogen in blue.

**Figure 3:**
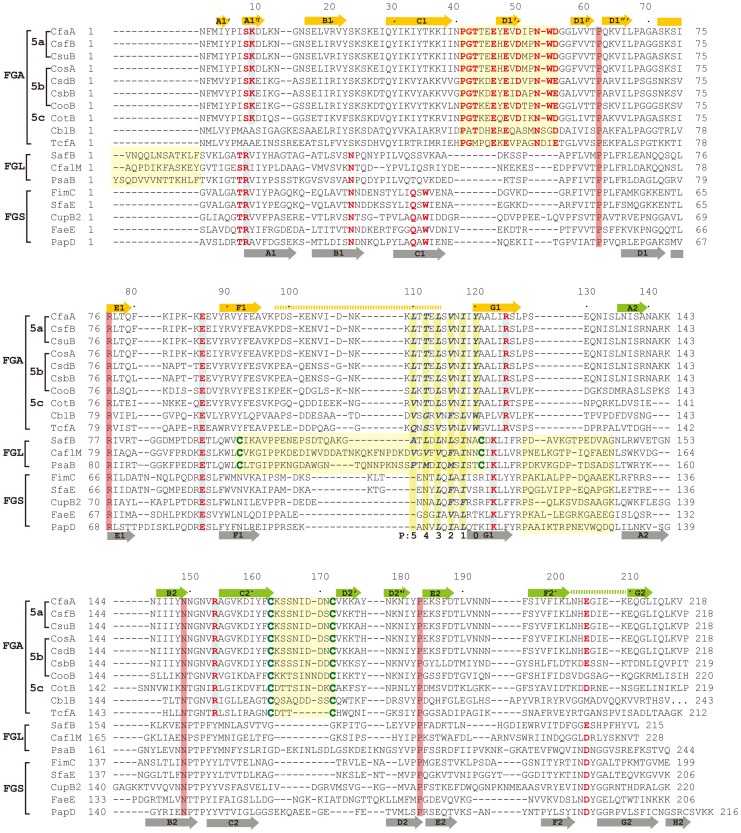
Structure-based sequence alignment of chaperone proteins involved in pilus assembly. This alignment is based on chaperone structures of CfaA of CFA/I fimbriae (this work), PapD of P pili (PDB:1QPX), FimC of type 1 pili (PDB: 1ZE3), SfaE of S-pili (PDB:1L4I), CupB2 of CupB pili (PDB: 3Q48), FaeE of F4 fimbriae (PDB:3F6L), SafB of Saf pili (PDB:2CO7), and Caf1M of F1 pili (PDB:2OS7). Other included sequences are PsaB of pH 6 fibril of *Y. pestis*, CsfB, CsuB, CosB, CsdB, CsbB, CooB, and CotB of Class 5 ETEC pili, and chaperones of Class 5 pili from other organisms such as CblA of Cbl pili from *B. cenocepacia* and TcfA of Tcf pili from *S. enterica*. The sequence of TcfA has 243 residues and its C-terminal tail is truncated to fit into the figure. The three sub-families of chaperones are referred to as FGA, FGL and FGS. The numbering of secondary structural elements is based on the structure of PapD [2] and are illustrated as gray arrows below the PapD sequence. β-strands for CfaA are shown as orange arrows for the N-terminal domain and green arrows for the C-terminal domain. Dashed orange and green lines indicate disorders in the structure of CfaA. Conserved residues in all chaperones are boxed in red. Conserved residues specific to each subfamily are highlighted in red. Insertions and extensions in each subclass are boxed in yellow. The hydrophobic residues in the alternating pattern of hydrophobic-hydrophilic residues in the donor strand prior to the G1 strand are in italic and boxed in yellow and are indicated at bottom of the alignment as positions P0 to P5. Cysteine residues that form disulfide bonds are highlighted in green and boxed in yellow.

CfaA and other chaperones of known ETEC Class 5 pili, all classified in the usher protein α clade [19], share high polypeptide sequence identity within this class (≥26%). By contrast, this group shares very low identities (≤15%, Table S3) with CUP chaperones of other fimbrial families, making accurate sequence alignment challenging. Availability of the atomic structures of chaperones from different clades enabled a structure-based sequence comparison. These structures include FaeE in κ [27], FimC and SfaE in γ1 [21], Caf1M and SafB in γ3 [5], [20], [23], CupB in γ4 [22], and PapD in π clade [2]. Using the CfaA structure reported here, a structure-based sequence alignment of Class 5 fimbrial chaperones with those of the other families (Figs. 2A and 3) reveals greater conservation in the N-terminal domain, which serves as the subunit-binding region and participates in subsequent donor-strand exchange, than the C-terminal domain, which is thought to be responsible for usher recognition [20].

In CfaA and all other Class 5 chaperones, two structural features are shared with the FGL chaperones (Table 2). First, the F1–G1 subunit-interacting loop is long, consisting on average of 20 residues, distinguishing it from the much shorter loops of the FGS chaperones (Fig. 3). Second, the subunit-binding motif immediately preceding the G1 strand features at least four candidate subunit-interacting hydrophobic residues (L/V114, V/F116, I/L118, Y/W120) rather than three in the FGS family (Fig. 3). This block of alternating hydrophobic-hydrophilic residues is, however, shifted by two residues towards the C-terminus in comparison to both FGL and FGS family chaperones. It is remarkable that Class 5 chaperones also share two features in common with FGS chaperones (Table 2). First, like FGS chaperones the Class 5 chaperones lack an N-terminal extension preceding the N-terminal A1 strand that is essential for subunit binding by FGL chaperones [28] (Fig. 3). Second, both the FGS and Class 5 chaperones lack the disulfide bridge that stabilizes the F1–G1 loop, which is conserved in the FGL chaperones (Fig. 3) and shown to be critical to formation of the FGL chaperone-subunit complex [29], [30].

**Table 2:** Unique features and possible functions for each subfamily of chaperones.

Characteristic features	FGL	FGS	FGA	Proposed functions of unique features (Mutations introduced in CfaA)
Average length of the F1–G1 loop	24 (long)	13 (short)	20 (long)	
No. required hydrophobic residues in donor strand	4	3	4	Binding cognate pilin subunit (L114A/V116A/I118A/Y120A)
N-terminal extension (12–14 residues)	Yes	No	No	
Disulfide bond between F1–G1 strands	Yes	No	No	
D1′ insertion	No	No	Yes	Pilin or usher interaction (T44A/E45A/E46A)
C2–D2′ insertion	No	No	Yes	Pilin or usher interaction (K164-N171; 8×A)
Disulfide bond in C2–D2′ loop	No	No	Yes	Stabilizing C2–D2′ insertion (C163S/C172S)
Length of peptide linker between two domains	14 (long)	14 (long)	7 (short)	
Residue for subunit anchoring	R20^a^	R8^b^	R154	Pilin subunit interaction (K9A/E86A/R125A/R154A)

a R20 is for Caf1M of *Y. pestis*.

b R8 is for PapD of P pili.

Importantly, the Class 5 chaperones also possess several structural features that are absent in both the FGL and FGS chaperones (Table 2). They contain an insertion (D1′ insertion) that includes the D1′ β-strand and is rich in acidic residues (E45, E46, E48, D50 and D55) (Figs. 1A and inset), which form several pairs of salt bridges with contiguous basic residues (K36, R90 and R185). All Class 5 chaperones contain a long, very hydrophilic insertion in the C2–D2′ loop (K164 to N171, C2–D2′ insertion) that is stabilized by a unique disulfide bond (C163–C172) (Figs. 1A and 3). The linker between N- and C-terminal domains of Class 5 chaperones is considerably shorter than those for FGL and FGS chaperones (Fig. 3). In the Class 5 chaperones, there is no readily apparent proxy for a conserved N-terminal basic residue in the FGL (e.g., R20 in Caf1M) and FGS (e.g., R8 in PapD) chaperones that is required for anchoring of the cognate pilin subunit through interaction with its C-terminus [6], [31]. The side chain of the corresponding K9 residue in CfaA points away from the chaperone cleft, disfavoring potential contact with a bound subunit (Fig. 2C). In the two members of Class 5 chaperones not from ETEC, CblA and TcfA, the equivalent lysine residue is absent. Evidence is provided below to suggest that this anchoring function is served by R154 in CfaA, a residue that is conserved in all Class 5 and absent in FGS and FGL chaperones (Fig. 3). Given these multiple distinctions, we propose that the Class 5 chaperones be placed into a separate family distinct from the FGL or FGS chaperones.

### Four interacting hydrophobic residues are needed in the donor strand in Class 5 family chaperones

**Figure 4:**
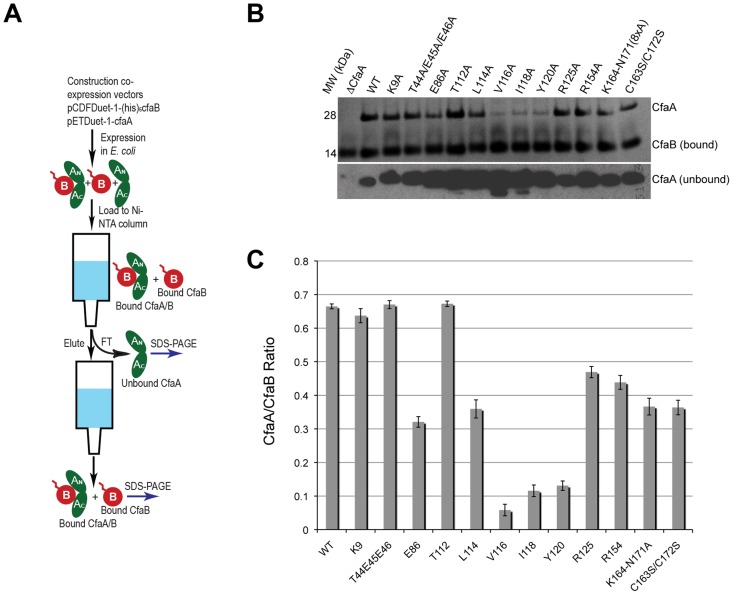
Characterization of CfaA mutants in forming complex with CfaB. (A) Schematic description of the procedure for preparing CfaA/B complex. (B) SDS-PAGE of eluted fractions from Ni-NTA affinity column for wild-type CfaA/B complex and various CfaA mutants detected by Coomassie Brilliant Blue staining. From lane 1 to 14 are CfaB co-purified with CfaA variants: No CfaA, wild type CfaA, K9A, T44A/E45A/E46A, E86A, T112A, L114A, V116A, I118A, Y120A, R125A, R154A, K164-N171(A×8) and C163S/C172S. Unbound CfaA in flow-through fractions were also subjected to SDS-PAGE analysis and detected by immunoblot with specific antibody against CfaB. (C) Ratios between CfaA variant and CfaB were obtained from densitometry analysis averaged over six independent SDS-PAGE experiments shown in (B). Error bars are indicative of standard deviations among independent experiments.

**Figure 5:**
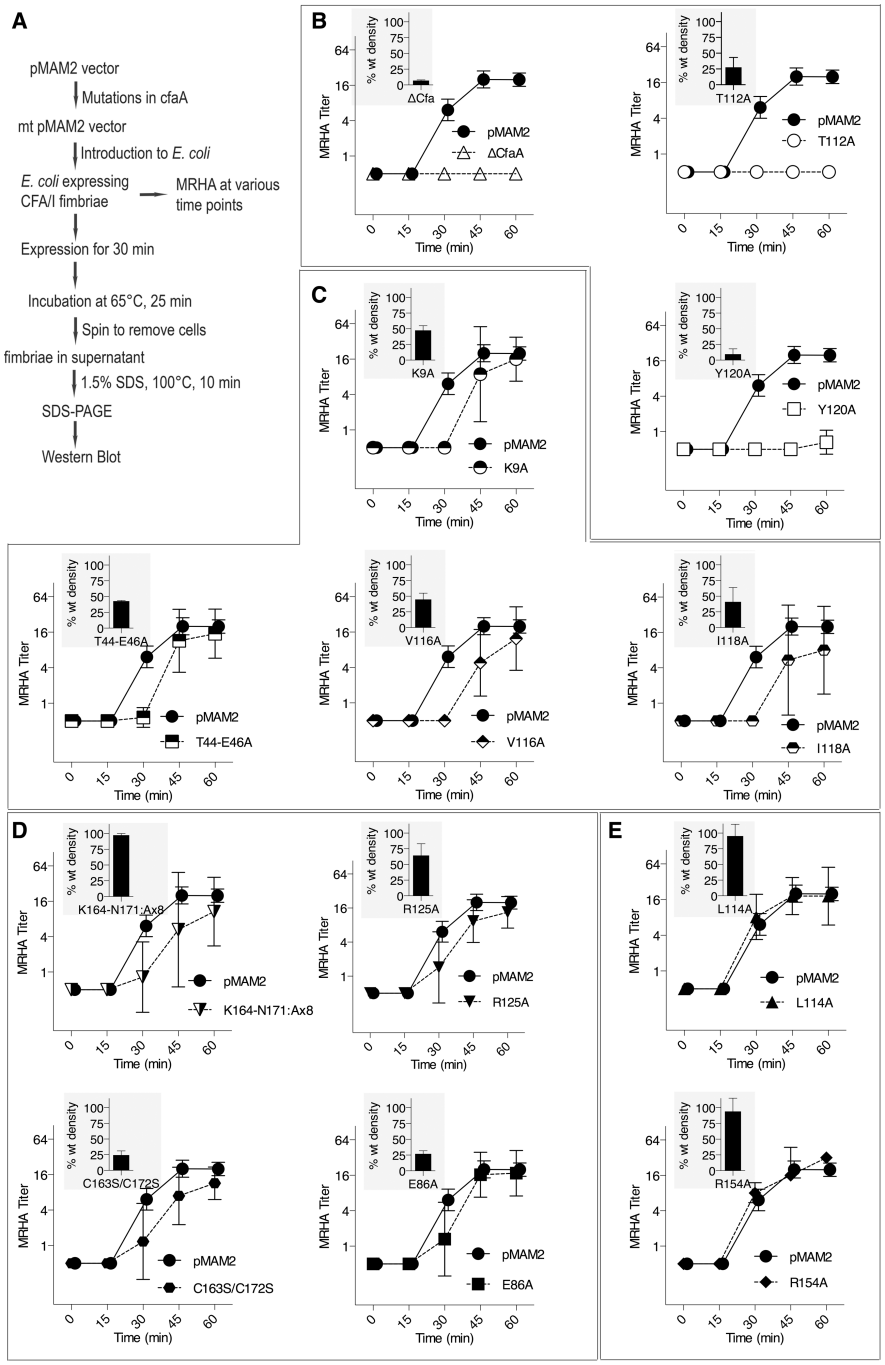
Effect of CfaA mutations on pilus pilus formation. (A) Schematic description of the procedures followed to determine the amount of CFA/I pili on the surface of *E. coli* harboring the CFA/I operon with either wild type (pMAM2) or modified CfaA. (**B–E**) Paired graphs are shown for each mutant, showing the rate of acquisition of functional pili as determined by the MRHA assay (fp_rate_) and (shaded graph insert in the upper left corner) the relative amount of surface-extracted CfaB after 30 minutes induction (piliation at 30 minutes [p30]). (**B**) A negative-control ΔCfaA mutation and two other modifications result in complete abolition of MRHA expression through 60 min of pilus induction and a correspondingly marked reduction in p30. (**C**) Four CfaA mutations exhibited delayed fp_rate_, all of which also showed a lower p30 in comparison to that associated with wild type CfaA. (**D**) Four CfaA mutations exhibited equivocal results with one (CfaA/K164-N171:A×8) showing a lag in fp_rate_ compared to pMAM2 but wild type p30 levels; and the other three (R125A, E86A, and C163S/C172S) showed an fp_rate_ not significantly lower than CfaA wild type over time, but a p30 lower than that of wild type. (E) Two mutants (L114A, R154A) exhibited both an fp_rate_ and p30 that was not different from the CfaA wild type. MRHA titers are graphed as geometric mean titers ±95% confidence intervals. Anti-CfaB Western blot data at 30 minutes (p30) was graphed as the relative amount ([density mutant/density pMAM2]×100 ± SEM) of surface-extracted CfaB.

Structure-based sequence alignment revealed a number of distinct features of Class 5 chaperones. To investigate the role of each of these unique structural attributes in subunit refolding, stabilization, escort function and usher interaction, mutations were introduced into each region with subsequent phenotypic analysis of the modified CfaA chaperone. While the ability of CfaA to stabilize the CfaB major subunit in an assembly-competent state was assessed using a pull-down assay and expressed as CfaA/CfaB ratio (Fig. 4), the assay that measures the amount of surface pili and the time-dependent mannose-resistant hemagglutination (MRHA) assay were used to reveal impairment of CfaA function in pilus assembly with respect to subunit transport and usher interaction (Fig. 5A). Accumulation of surface pili was determined after 30 minutes of induction by comparison of the amount of pili extracted from the bacterial surface by heat treatment (piliation at 30 minutes, p30) followed by SDS PAGE and anti-CfaB Western blot analysis (Fig. 5). As a control for periplasmic leakage of CfaB during heat extraction, anti-CfaA Western blots were also performed on these preparations with nominal detection of the periplasmic chaperone (data not shown). For recombinant *E. coli* containing the CFA/I operon with a native or modified CfaA gene, the functional pilus assembly rate (fp_rate_) was determined by induction of CFA/I expression and performance of a semiquantitative MRHA assay at 15-minute intervals over an hour (Fig. 5).

Between F1 and G1 β-strands of all chaperones, there is a stretch of peptide with alternating hydrophobic-hydrophilic residues (Fig. 3). The FGS and FGL chaperones feature three and five hydrophobic residues, respectively. Each of these hydrophobic residues is assigned a position as P1, P2, P3, P4 or P5 based on its interaction site on the pilin subunit [32] (Figs. 2B and 3). Like FGL, Class 5 chaperones are predicted to have a minimum of four hydrophobic residues in the donor strand, but their positions are shifted compared to FGL chaperones based on the structure-based sequence alignment (Figs. 2A, 2B and 3). In keeping with the original convention [32], the hydrophobic residues L114, V116 and I118 would correspond to positions P3, P2, and P1, respectively, based on the alignment, leaving no assignment for Y120. Thus, we propose to assign Y120 the P0 position, which is a site unique to Class 5 chaperones as it relates to subunit interaction (see below). It should be noted that there is a hydrophilic residue (T112) at the P4 position, and a hydrophobic residue (L110) at the P5 position (Fig. 3). These two positions are not all conserved beyond the chaperones in the 5a and 5b subclasses (Fig. 3).

To assess the contribution of each of these residues to subunit binding, the four pilin-interacting, hydrophobic residues (L114, V116, I118 and Y120) in the donor strand preceding the G1 β-strand were each modified to alanine. Additionally, a T112A mutation was also made. Except for T112 at the P4 position, individual alanine mutations of all hydrophobic residues led to a marked reduction in the CfaA/CfaB ratio from 8.7% to 54.0% (Figs. 4B and 4C), indicating the importance of each of these residues in forming a stable complex. The P0, P1, and P2 CfaA mutations (i.e., Y120A, I118A, and V116A, respectively) were each also associated with reduced p30 and fp_rate_ in comparison with native CfaA with most dramatic reduction for the Y120A mutant, indicating impaired bacterial surface piliation (Figs. 5B and 5C). These results are consistent with the pull-down experiments and confirm the mechanism by which the subunit maintaining its competency in assembly is largely by the hydrophobic interactions between the donor strand from chaperone and the binding groove of the subunit. The L114A substitution in CfaA at the P3 position resulted in a clear reduction in the CfaA/CfaB ratio (Figs. 4B and 4C), but no detectable reduction in bacterial fimbriation as determined by p30 and fp_rate_ experiments, respectively (Fig. 5E), suggesting that a change at P3 alone is not rate limiting with respect to downstream pilus assembly. The T112A substitution at the P4 position in CfaA did not decrease the CfaA/CfaB ratio (Figs. 4B and 4C), but was associated with a marked decrease in p30 piliation and no detection of MRHA activity over time (Fig. 5B), suggesting that this mutation negatively impacts CFA/I assembly without apparent effect on major subunit binding.

### Both C2–D2′ and D1′ insertions are required for CFA/I fimbriation

The Class 5 chaperones feature two distinct sequence insertions: the D1′ insertion in the N-terminal lobe and the C2–D2′ insertion in the C-terminal lobe (Figs. 1A and 3, Table 2). The C2–D2′ insertion is additionally stabilized by a conserved disulfide bond between C163 and C172 (Fig. 1A). To probe function of the C2–D2′ insertion, alanine mutations were introduced to a block of eight residues (from K164 to N171) in the insertion loop. Moreover, the class-specific disulfide bond (C163 and C172) connecting the ends of the loop was also changed by mutating the two cysteine residues to serine residues. Both mutants showed similar decreases in CfaA/CfaB ratio of 54.7% and 55.2%, respectively, for K164-N171A and C163S/C172S (Fig. 4B), suggesting that neither of these motifs is critical to CfaA's ability to stabilize CfaB subunit. Correspondingly, the two mutants by fp_rate_ showed a right shift wherein MRHA activity was lower than wild-type CfaA at 30 minutes with catch-up to wild-type CfaA levels by 45–60 minutes (Fig. 5D), even though they displayed different p30 piliation levels. These results suggest a role for the C2–D2′ insertion, especially the disulfide linkage, in either the upstream subunit interaction or the down stream pilus assembly or both.

The introduction of three mutations in the middle of the acidic D1′ insertion (T44A/E45A/E46A) did not alter the CfaA/CfaB ratio as compared to the wild type (Fig. 4), but did affect p30 piliation as well as fp_rate_ levels (Fig. 5C). Thus, the unique D1′ insertion of CfaA plays a role in pilus assembly.

### CfaA uses a different set of residues to anchor pilin subunit as apposed to FGL and FGS chaperones

Structure-based sequence alignment indicated that K9 of CfaA is offset by one residue from the conserved N-terminal arginine in the FGL and FGS family chaperones (Fig. 3). Structure superposition between PapD and CfaA seems to suggest that the function of this conserved arginine in FGL and FGS chaperones is replaced by R154 in CfaA (Figs. 1B and 2C). In addition to the N-terminal arginine residue, a conserved lysine residue in the G1 strand of FGL and FGS chaperones (K112 in PapD and K139 in Caf1M) was shown to assist subunit binding [6], [31], [33]. The equivalent of this conserved lysine residue in CfaA is R125, which interestingly is also offset in the sequence alignment (Figs. 1B, 2A, 2B and 3). The conformation of these residues appears to be stabilized by salt bridges to another conserved glutamate residue (E86 in CfaA, E83 in PapD and E92 in Caf1M, Figs. 2 and 3). The offset in sequence alignment and lack of conservation in CblB and TcfA sequences indicate that K9 in CfaA may not perform the same function as anchoring residues for subunit binding, as demonstrated experimentally for FGL and FGS chaperones. To verify this hypothesis, a K9A mutation was introduced into CfaA, which had no apparent effect on the stability of the CfaA/CfaB complex (Figs. 4B and 4C). We also made an R125A mutant, which resulted in a decrease in the CfaA/CfaB ratio by the pull-down assay (Figs. 4B and 4C). Both mutations were associated with lowed p30 piliation level, while K9A was also associated with a delayed fp_rate_ (Fig. 5D), suggesting some degree of impedance of pilus bioassembly with each of these mutations.

Structure superposition between PapD and CfaA suggested that the function of the N-terminal conserved arginine in FGL and FGS chaperones may be replaced by R154 in CfaA, which is only conserved in Class 5 chaperones (Figs. 2C and 3) and is stabilized by residue E86 via a salt bridge (∼2.7 Å). In fact, E86 is conserved in all families of chaperones (Fig. 3). To confirm this hypothesis, alanine substitutions to R154 and E86 were introduced. Both mutations were associated with a reduction in the ability of CfaA to stabilize CfaB (Figs. 4B and 4C), while the only apparent defeat in piliation associated with either of these mutations was a lower p30 piliation level for E86A (Figs. 5D and 5E). The divergent findings in the binding and piliation assays may be consistent with the interpretation that the formation of CfaA/CfaB complex is a process that is not coupled tightly to that of assembly.

## Discussion

### Class 5 chaperones represent a novel family distinct from chaperones in FGL and FGS families

Chaperone-subunit complexes were among the first fimbrial components for which crystal structures were determined [3], [5], [26], [27], [32]–[34]. These structures elucidated the donor-strand complementation (DSC) and exchange (DSE) mechanism, integral to the subunit stabilization and pilus assembly of CUP pili. One of the most important, general features of these chaperones is the essential interactions between the G1 strand and the hydrophobic groove of pilus subunits [26]. Beyond the observed commonalities, sequence and structural differences have been recognized for chaperones of different pili, leading to the subdivision of FGL and FGS family chaperones [6], [29]. It was also recognized that FGL chaperones were found only in pili having thin, flexible morphology, whereas FGS chaperones appear to only assist assembly of rod-like pili [15], [35], [36]. In this work, the crystal structure was determined for the CfaA chaperone of CFA/I pili, which represents the first atomic resolution chaperone structure for the Class 5 pilus family. On the basis of structure-based sequence alignment with FGS and FGL chaperones, Class 5 chaperones, as represented by CfaA, display unique features distinguishing them from both FGL and FGS families. Given the historical assignment of Class 5 pili to the alternate-chaperone pathway for assembly, we propose the designation of FGA (F1–G1 Alternate) chaperones for this family.

**Figure 6:**
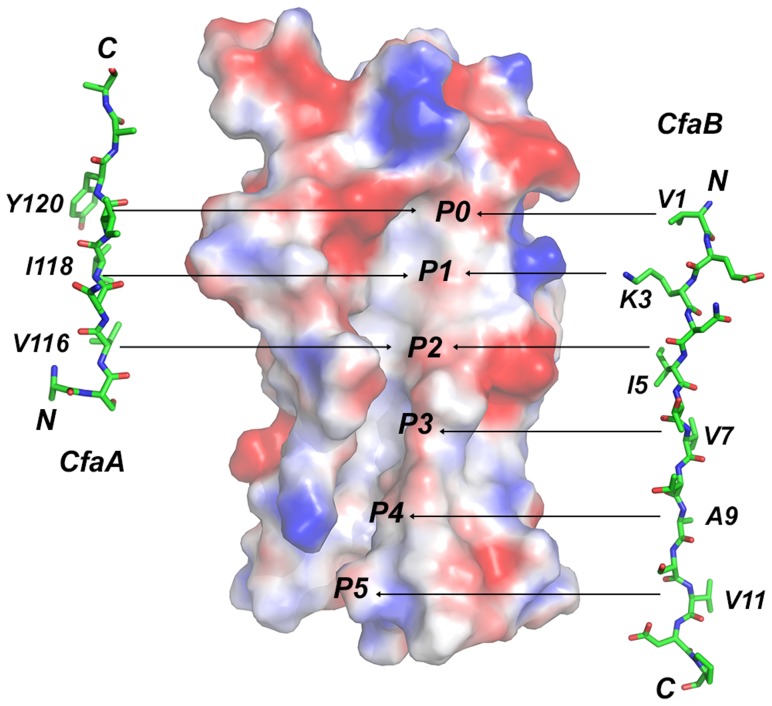
Comparison of donor-strand complementation by CfaB with that proposed by CfaA. Six hydrophobic pockets are shown in the donor-strand binding groove of the electrostatic potential surface of the pilin subunit CfaB in the absence of donor strand. The six pockets are sequentially labeled from P0 to P5. The structure of the CfaA donor strand as determined in this work is given to the left of the CfaB surface, whereas that of CfaB is shown on the right based on the donor-strand complemented CfaB structure [17]. Residues that are presumed to fit into these pockets are also indicated.

FGA chaperones bear certain similarities to both FGL and FGS chaperones, but also possess several structural and functional features that make them unique. Similar to FGL chaperones, FGA chaperones have a long subunit-interacting loop harboring four hydrophobic residues for subunit interaction. FGA and FGS chaperones both lack an N-terminal extension and the disulfide bridge that stabilize the F1–G1 loop for FGL chaperones. Based on structure-based sequence alignment and the mutational analyses presented herein, there are unique structural features that are also important for FGA chaperone function (Table 2). First, the four subunit-interacting hydrophobic residues in the F1–G1 loop, designated as P0–P3, are shifted in position by two residues towards the C-terminus (Figs. 3 and 6). Second, CfaA appears to use a different set of residues (R154 and E86) to anchor the subunit into the binding cleft. Third, it features two insertions, a D1′ insertion in the N-terminal domain and a C2–D2′ insertion stabilized by a disulfide bridge in the C-terminal domain, which may play a role either in pilus bioassembly or in major pilin interaction (Figs. 4 & 5).

Supporting evidence for the designation of FGA family chaperones also comes from the sequence alignment from two FGA chaperones that are not part of Class 5 ETEC (Fig. 3). One is CblA from Cbl pili of *Burkholderia cenocepacia* and the other is TcfA of Tcf pili from *Salmonella enterica*. In these two sequences not only are all the unique features to FGA chaperones preserved but also the N-terminal SK motif is no longer present, whose function is, as proposed, replaced by R154 that indeed is conserved only in FGA family. Furthermore, the P0 position features an aromatic tryptophan residue for these two members of the FGA chaperone. Phylogenetic analyses of the usher proteins for CUP fimbriae found that all Class 5 pili fall into a single α-clade [19], corroborating their prior classification into the distinct group of pili assembled by the alternate chaperone pathway based on their genetically distinct chaperones [14].

### Implications for CfaA-subunit interactions and pilus assembly

In this work, mutations were introduced to residues and motifs of the CfaA chaperone, which are unique to the FGA family chaperones based on the structure-based sequence alignment. The effects of these mutations on CfaA function as it relates to stabilizing the major pilin subunit CfaB in an assembly-competent state and to pilus assembly were examined (Figs. 4 and 5, Table 3). Based on the pull-down assay, mutations in CfaA either dramatically reduced the CfaA/CfaB ratio (V116A, I118A and Y120A), showed no effect (for example K9A, T44A/E45A/E46A and T112A) or displayed moderate reduction in the CfaA/CfaB ratio (Figs. 4B and 4C). On the basis of their effects to pilus assembly, these mutations can also be categorized into four groups. One group contains mutations (T112A and Y120A) that showed little piliation and no detectable MRHA (Fig. 5B), while a second group (L114A and R154A) showed no effect in both (Fig. 5E). A third group (K9A, T44-E46A, V116A, and I118A) displayed a reduced p30 and, correspondingly, a significant delay in pilus assembly when compared to the wild-type CfaA (Fig. 5C). Finally, mutants (K164-N171A, R125A, C163/C172A, and E86A) in the fourth group exhibted equivocal results of mismatching p30 and fp_rate_ (Fig. 5D).

**Table 3:** Summary of effects of CfaA mutations on its interactions to CfaB subunit and pilus formation.

		Fimbriation		
Mutation Mean	CfaA/CfaB ratio	By heat extraction (p30)	By time-resolved MRHA (fp_rate_)	Proposed function
Wild type	0.66	100	-	-
K9A	0.64	45	Delayed^a^	Function partially replaced by R154
T44A/E45A/E46A	0.67	43	Delayed^a^	D1′ insertion, interacting with either subunit or usher
E86A	0.32	25	Minimal effect	Stabilizing R154
T112A	0.67	25	No fimbriation^b^	P4: donor-strand exchange
L114A	0.36	95	No effect	P3: donor-strand complementation
V116A	0.06	43	Delayed^a^	P2: donor-strand complementation
I118A	0.11	41	Delayed^a^	P1: donor-strand complementation
Y120A	0.13	10	No fimbriation^b^	P0: donor-strand complementation
R125A	0.47	65	Minimal effect	Interaction with either subunit or usher
R154A	0.44	95	No effect	Replacing K9 for subunit interaction
C163S/C172S	0.36	25	Minimal effect	Disulfides stabilizing C2–D2′ loop.
K164-N171 (8×A)	0.37	95	Delayed^a^	C2–D2′ insertion for either subunit interaction or pilus assembly
ΔCfaA	0.0	5	No fimbriation	

a Wild type MRHA titer by 45 min.

b Minimal to no MRHA detected through 60 min.

It should be noted that the pull-down assay (CfaA/CfaB ratio) measures only the stability of the CfaA/CfaB complex in solution; it does not provide information on how CfaA or its mutants interact with CfaE, the minor pilin subunit, nor CfaC, the usher. Piliation by p30 measures the amount of CfaB on the bacterial surface but is unable to differentiate between the wound and unwound forms of CFA/I pili [17]. The time-dependent MRHA (fp_rate_) estimates the level of functional surface pili semiquantitatively. Not surprisingly, effects demonstrated by the pull-down and piliation assays are not necessarily correlated, suggesting the following possibilities: (1) Mutant CfaA altered interactions with the minor adhesin CfaE or the usher CfaC instead of with CfaB. (2) CfaA mutations could affect only the on-rate but not the off-rate of its interaction to CfaB. The on-rate is not measured by the pull-down assay because the dissociation of the CfaA/CfaB heterodimer is irreversible. And (3) the formation of CfaA/CfaB complex is a process that is not tightly coupled to the pilus assembly. In reality, each mutation in CfaA may contribute to all these possibilities. An example is the donor-strand T112A mutation that had no apparent effect on the stability of CfaA/CfaB complex but appeared to abolish piliation. A similar conclusion could be made for the L114A and R154A mutations that led to less stable CfaA/CfaB complex but wild type levels of piliation.

Previously, it was reported that besides the general hydrophobic interactions provided by the donor strand, all chaperones that assist pilus assembly have conserved “critical basic residues” in the substrate binding cleft, which interact with the C-terminal residue of a bound subunit, any mutations in those basic residues invariably affect pilus assembly [31]. Although CfaA and related FGA chaperones also have the pair of conserved basic residues, K9 and R125 in CfaA, corresponding to those in FGL and FGS chaperones, structure-based sequence alignment showed an offset in the alignment by one residue (Fig. 3). Moreover, in the CfaA structure the side chain of K9 points away from the cleft and is distant from R125, making it unlikely to interact with pilin subunit (Fig. 2C). Indeed, our mutational analyses support this conclusion.

Based on the crystal structure of CfaA, we suggest that R154, which is stabilized by the conserved E86, serves the anchoring function carried out by residue K9 in the FGS and FGL chaperones. Consistent with this hypothesis, the R154A mutation in CfaA results in a reduction in the stability of the CfaA/CfaB complex (Figs. 4B and 4C). However, both piliation assays, p30 and fp_rate_, detected comparable amount of surface pili for the R154A mutant to that of wild type (Fig. 5C), suggesting that either R154A mutation alters the capture of CfaB by CfaA during CfaA-assisted subunit refolding in periplasm or the rate-limiting step in the pilus assembly is at the site of usher protein.

The observation that the FGA chaperones have donor strand residues (P0–P3) shifted in position by two residues suggests that the bound subunit may fit deeper into the chaperone cleft (Figs. 3 and 6), leading to the speculation that this altered pattern of interaction could be a source of specificity between cognate partners. The two hydrophilic residues flanking the hydrophobic stretch in donor strand (T112 and R125) are perhaps important for the donor-strand exchange function at the pilus assembly site [5], as mutations at these sites either destroyed or diminished piliation but had little impact to the stability of the chaperone-pilin complex in solution.

In summary, the elucidation of unique structural and functional features in the CfaA chaperone of CFA/I fimbriae provides a clear case for separating Class 5 chaperones into a distinct group of periplasmic chaperones, which are distinguished from those in the FGL and FGS families. Mutations introduced into these unique features of FGA chaperones produced effects that are indicative of their roles in cognate subunit recognition and in pilus assembly. The question remains unresolved as to how CfaA is able to recognize and interact with both the minor (CfaE) and the major (CfaB) CFA/I pilus subunits, which requires further structural and functional investigations.

## Materials and Methods

### Cloning and mutagenesis

The plasmid pNTP513 [37] was used as a template for PCR amplification of the coding regions of mature CfaB (residues 24–170), using primers containing *Nde*I and *Xho*I restriction sites at 5′- and 3′-end, respectively (Table S2). The digested PCR product was cloned into a pCDFDuet-1 vector (Novagen) with an added hexahisidine tag N-terminally to the mature CfaB to yield the plasmid pCDFDuet-1-*(his)_6_cfaB*. The CfaA gene was also amplified from pNTP513 and cloned into an expression vector pET24a (Novagen) with an added hexahistidine tag at C-terminus, yielding the vector pET24a-*cfaA(his)_6_*. CfaA (20–238) was also cloned into the pETDuet-1 vector (Novagen) without modification to yield the vector pETDuet-1-*cfaA*. The CFA/I operon (*CfaABCE*) expression plasmid pMAM2 construction has been described previously [16].

Site-specific mutations were introduced to pETDuet-1-cfaA and pMAM2 using site-directed mutagenesis kit (New England Biolab), yielding the following vectors: pETDuet-1-*cfaA(K9A)* and pMAM2(*cfaA:K9A*), pETDuet-1-*cfaA*(*T44/E45A/E46A*) and pMAM2(*cfaA:T44/E45A/E46A*), pETDuet-1-*cfaA*(*E86A*) and pMAM2(*cfaA:E86A*), pETDuet-1-*CfaA(T112A)* and pMAM2(*cfaA:T112A*), pETDuet-1-*cfaA(L114A)* and pMAM2(*cfaA:L114A*), pETDuet-1-*cfaA(V116A)* and pMAM2(*cfaA:V116A*), pETDuet-1-*cfaA(I118A)* and pMAM2(*cfaA:I118A*), pETDuet-1-*cfaA(Y120A)* and pMAM2(*cfaA:Y120A*), pETDuet-1-*cfaA(R125A)* and pMAM2(*cfaA:R125A*), pETDuet-1-*cfaA(R154A)* and pMAM2(*cfaA:R154A*), pETDuet-1-*cfaA*(K164-N171:A×8) and pMAM2(*cfaA:K164-N171:A*×8), and pETDuet-1*-cfaA(C163S/C172S)* and pMAM2(*cfaA:C163S/C172S*).

An inframe deletion of amino acids 15–222 of *cfaA* was introduced to pMAM2 using QuikChange II XL Site-Directed Mutagenesis Kit (Agilent Technologies), resulting in pMAM2(*ΔcfaA*).

### Expression and purification of CfaA(his)_6_

To express hexahistidine-tagged CfaA(his)_6_, the expression plasmid pET24a-*cfaA(his)_6_* was transformed into *E. coli* BL21(DE3) strain. *E. coli* cells were grown in terrific broth (Research Products International Corp.) in the presence of 50 µg/ml of kanamycin at 37°C. When cell density reached 0.8 at OD_600_, expression of recombinant proteins was induced by adding isopropyl β-D-1-thiogalactopyranoside (IPTG) to 0.8 mM. After a further 16 hours of incubation at 18°C, cells were collected by centrifugation.

Cell pellets were resuspended in a hypertonic buffer containing 60 mM Tris-HCl, pH 7.5 and 20% glucose for 10 minutes followed by another centrifugation. Periplasmic extracts were prepared by resuspending cell pellets in an ice cold hypotonic buffer consisting of 5 mM MgCl_2_, 5 mM CaCl_2_, 20 mM Tris-HCl, pH 7.5, and 50 mM NaCl followed by a high-speed centrifugation at 16,000× g for 30 min. The supernatant was loaded onto a Ni-NTA superflow column (Qiagen) pre-equilibrated with a binding buffer (20 mM Tris-HCl, pH 7.5, and 100 mM NaCl) plus 20 mM imidazole. After washing the resin with 5 column volumes of binding buffer plus 30 mM imidazole three times, CfaA(his)_6_ was eluted with the same binding buffer plus 300 mM imidazole. As a last step, size exclusion chromatography with a Superdex 200 column (GE Healthcare Life Science) was used to further purify CfaA(his)_6_ and the resulting protein was concentrated to 10 mg/ml for crystallization using an Amicon Ultra-15K with10 kDa MW cutoff concentrating device (Millipore).

### Formation of CfaA/B complex and purification of CfaA variants complexed with CfaB

*E. coli* BL21(DE3) was co-transformed with pCDFDuet-1-*(his)_6_cfaB* and one of the following additional plasmids: pETDuet-1 vector (negative control), pETDuet-1-*cfaA* (positive control), and each of the vectors above containing the specified mutation in cfaA. These co-transformants were grown at 37°C in LB media supplemented with 50 µg/ml each of streptomycin sulfate and ampicillin. When the culture reached an OD_600_ of 0.8, IPTG was added to a final concentration of 0.8 mM to induce expression, with subsequent incubation for 16 hours at 18°C, at which point cells were collected by centrifugation.

Periplasmic extract was prepared from each co-transformant in a manner identical to that described above for BL21(DE3)/pET24a-*cfaA(his)*_6_ and loaded onto a Ni-NTA superflow columns (Qiagen) pre-equilibrated with the binding buffer supplemented with 20 mM imidazole. The flow-through was collected for analysis of unbound CfaA. The columns were then washed 3 times with 5 column volumes of binding buffer supplemented with 30 mM imidazole, with subsequent elution with the binding buffer adjusted to an imidazole concentration of 300 mM. The eluate was analyzed for the presence of CfaA/(his)_6_CfaB complexes.

Flow-through and eluate samples were subjected to SDS-PAGE. Samples were heated to 70°C for 3 min, loaded and separated on 12% Bis-Tris polyacrylamide gels (Invitrogen). Eluate samples were analyzed after staining by coomassie blue. Recovered amounts of CfaA and (his)_6_CfaB for each of the co-transformants with modified CfaA were compared to the control co-transformant (unmodified CfaA) to determine the relative amount of CfaA bound to (his)_6_CfaB (complex formation). The flow-through samples were transferred to nitrocellulose for Western blot analysis using CfaA antiserum (1∶5000 dilution) to determine the relative amounts of expressed CfaA.

### Analysis of CFA/I Fimbriation by mannose-resistant hemagglutination and heat extraction assay

The pMAM2 parent plasmid and each of the derivatives bearing a modified CfaA gene were transformed to the *E. coli* host strain BL21-AI (Invitrogen), which places the CFA/I fimbrial operon under the control of an arabinose-inducible T7 promoter. These strains were grown in LB media with kanamycin (50 µg/ml) at 30°C. When the culture density reached an OD_600_ of ≥0.5, CFA/I fimbrial expression was induced with addition of arabinose to a final concentration of 0.2%. At 0, 15, 30, 45, and 60 minutes after induction at 30°C, cells were collected by centrifugation and resuspended in phosphate buffered saline with 0.5% D-mannose to a final OD_650_ of 40.

In a 12-well ceramic tile plate, 25 µl each of the bacterial suspension and 50 µl of a 1.5% bovine erythrocyte suspension were added to each well, and the plates were incubated with rocking on ice for 20 minutes. Positive mannose-resistant hemagglutination (MRHA) was determined visually by observation of any degree of erythrocyte clumping. For each bacterial preparation that gave a positive MRHA reaction with addition of the initial bacterial suspension (i.e., OD_650_ = 40), a two-fold dilution series was performed using PBS with D-mannose as the diluent, and the dilution series was assayed for MRHA. The highest bacterial dilution yielding a positive MRHA reaction was recorded as the MRHA titer. All bacterial samples were tested in 4–5 separate experiments on different days, and each experiment was performed in duplicate.

Quantitation of surface-localized fimbriae by heat extraction of bacteria was performed concomitantly with the aforementioned MRHA experiments. One ml of each concentrated suspension of bacteria (i.e., OD_650_ = 40) was removed at the 0, 30, and 60 min time points, pelleted by centrifugation and resuspended in 250 µl PBS. After incubation at 65°C for 25 min, cells were removed by centrifugation at 6,000× g for 30 min. These heat extract preparations were placed in sample buffer containing 1.5% SDS and placed at 100°C for 10 min just prior to separation by SDS-PAGE (15% polyacrylamide). After transfer to nitrocellulose, Western blot analysis was performed by chemiluminescence using mouse antiserum (at 1∶5,000,000 dilution) against recombinat CfaEB [17] and the SuperSignal West Femto Complete Mouse IgG Detection kit (Pierce). Western blot analyses were similarly performed using anti-CfaA antiserum (at 1∶1,000,000 dilution) to monitor for leakage from the periplasmic space.

### CfaA crystallization and structure determination

Purified CfaA(his)_6_ was crystallized by the hanging drop vapor diffusion method at 293 K, mixing 2 µl of protein (10 mg/ml) with 2 µl of well solution containing 22% PEG3350, 0.2 M NaCl and 0.1 M MES pH 5.3. The platinum and lead derivatives were prepared by soaking native crystals in well buffer supplemented with 2 mM K_2_PtCl_4_ and 15 mM Pb(CH_3_COO)_2_, respectively, overnight. CfaA crystals were cross-linked using glutaraldehyde before flash-cooled in liquid propane in the presence of 30% glycerol [38].

Diffraction data sets were recorded at the SER-CAT BM beamline at the Advanced Photon Source (APS), Argonne National Laboratory (ANL) with a MAR-225 CCD detector. The data were integrated and scaled using the *HKL2000* package [39]. The structure was solved by the multiple isomorphous replacement coupled with anomalous scattering (MIRAS) method using the program suite PHENIX [40].

An initial CfaA model generated from SOLVE/RESOLVE [41] was manually completed in Coot [42], and was refined against a 1.9 Å resolution data set using REFMAC5 [43] from the CCP4 suite [44]. Multiple structure-based alignments were done in O [45]. The structure was validated using Molprobity [46].

### Coordinates

Atomic coordinates of the refined structures have been deposited in the Protein Data Bank (www.pdb.org) with the pdb code 4NCD for the structure of CfaA.

### Accession numbers

Proteins used in this study have the following accession numbers in the UniProtKB/SwissProt database: CfaA, E3PPC3; PapD, P15319; FimC, P31697 ; CooD, D7GKP2 ; CooB, P25731; CooA, P0ABW7; CooC, D7GKP1; CfaE, P25734; CfaB, E3PPC4; Caf1M, P26926; FaeE, P25401; SafB, Q93IN9; CupB, H3SUK7; CupB2, H3SUK8; SfaE, Q9EXJ6; PsaB, P69965; CsfB, Q93G70; CsuB, Q5SGF0; CosB, Q6R591; CsdB, Q5SGE5; CsbB, Q5SF91; CotB, Q47116; HifB, P45991; F17a, O30925; FasB, Q46992; CblB, B4ELG1; TcfA, S5GUW7.

## Supporting Information

Figure S1 **Ribbon diagram of dimeric CfaA related by crystallographic two-fold symmetry and the enlarged capping interface formed by hydrogen bonding between two subunit interacting loops.**
(TIF)

Table S1 **H-bonding interactions between residues from N- and C-terminal domains of CfaA.**
(DOC)

Table S2 **Water mediated H-bonding interactions between N- and C-terminal domains of CfaA.**
(DOC)

Table S3 **Comparison of amino acid sequences of periplasmic chaperones showing proportion of identical residues (unshaded; upper right) and similar (shaded; lower left) over entire length of precursor protein.**
(DOC)

Table S4 **Primers used in the work.**
(DOC)
