# Supplementary figures and images for: Structure of CfaA Suggests a New Family of Chaperones Essential for Assembly of Class 5 Fimbriae
Source: PLoS Pathog. 2014 Aug 14;10(8):e1004316. doi: 10.1371/journal.ppat.1004316 (PMC4133393; doi:10.1371/journal.ppat.1004316)

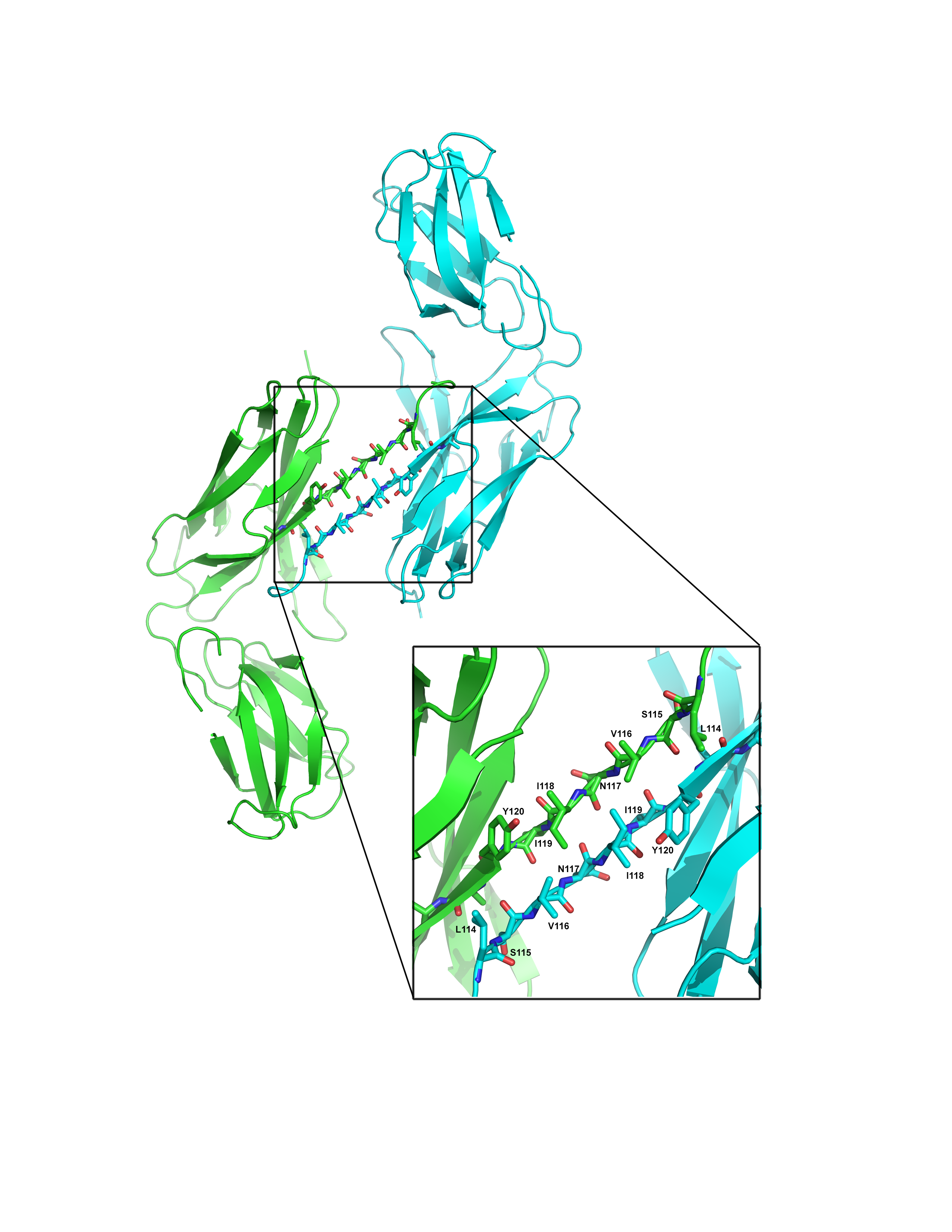

Supplement: Figure S1 — Ribbon diagram of dimeric CfaA related by crystallographic two-fold symmetry and the enlarged capping interface formed by hydrogen bonding between two subunit interacting loops. (TIF) [file ppat.1004316.s001.tif]
