# Supplementary material for: Structure of CfaA Suggests a New Family of Chaperones Essential for Assembly of Class 5 Fimbriae
Source: PLoS Pathog. 2014 Aug 14;10(8):e1004316. doi: 10.1371/journal.ppat.1004316 (PMC4133393; doi:10.1371/journal.ppat.1004316)
Supplement: Table S1 — H-bonding interactions between residues from N- and C-terminal domains of CfaA. (DOC) [file ppat.1004316.s002.doc]

**Table S1. H-bonding interactions between residues from N- and C-terminal domains of CfaA**

| **N-domain residue** | **Atom^a^** | **Distance (Å)** | **Atom** | **C-domain residue** |
| --- | --- | --- | --- | --- |
| E86 | N | 2.8 | O | N152 |
| G42 | O | 3.1 | NZ | K179 |
| K85 | NZ | 2.8 | OE2 | E184 |
| R125 | NH1 | 3.5 | OE2 | E205 |
| a - Atom name designations are according to the PDB convention: N, O are main chain nitrogen and oxygen atoms, respectively. NZ is the terminal amine nitrogen of lysine. ND2 is the amide nitrogen for asparagine. NH1 and NH2 are the two terminal guanidinium nitrogen atoms for the arginine residue, respectively. OG is hydroxyl oxygen of serine. OE2 is the carboxylate oxygen of glutamate. | | | | |
