# Supplementary material for: Structure of CfaA Suggests a New Family of Chaperones Essential for Assembly of Class 5 Fimbriae
Source: PLoS Pathog. 2014 Aug 14;10(8):e1004316. doi: 10.1371/journal.ppat.1004316 (PMC4133393; doi:10.1371/journal.ppat.1004316)
Supplement: Table S3 — Comparison of amino acid sequences of periplasmic chaperones showing proportion of identical residues (unshaded; upper right) and similar (shaded; lower left) over entire length of precursor protein. (DOC) [file ppat.1004316.s004.doc]

**Table S3. Comparison of amino acid sequences of periplasmic chaperones showing proportion of identical residues (unshaded; upper right) and similar (shaded; lower left) over entire length of precursor protein.**

|  | CstA | CfaA | Caf1M | HifB | F17a-D | PapD | FasB | FimC | FaeE | SfaE | CupB2 | SafB |
| --- | --- | --- | --- | --- | --- | --- | --- | --- | --- | --- | --- | --- |
| CstA | - | .10 | .33 | .18 | .23 | .17 | .14 | .21 | .20 | .24 | .19 | .28 |
| CfaA | .22 | - | .12 | .10 | .08 | .12 | .15 | .07 | .08 | .10 | .10 | .09 |
| Caf1M | .54 | .24 | - | .25 | .24 | .20 | .16 | .29 | .20 | .30 | .25 | .34 |
| HifB | .36 | .26 | .40 | - | .39 | .23 | .20 | .29 | .22 | .30 | .31 | .21 |
| F17a-D | .35 | .25 | .41 | .55 | - | .25 | .21 | .28 | .19 | .31 | .33 | .26 |
| PapD | .30 | .28 | .37 | .38 | .42 | - | .21 | .27 | .20 | .29 | .28 | .20 |
| FasB | .32 | .29 | .33 | .36 | .40 | .41 | - | .20 | .19 | .22 | .20 | .20 |
| FimC | .32 | .24 | .42 | .44 | .46 | .43 | .42 | - | .23 | .61 | .25 | .28 |
| FaeE | .34 | .22 | .36 | .38 | .40 | .40 | .39 | .40 | - | .23 | .21 | .15 |
| SfaE | .38 | .28 | .45 | .43 | .47 | .44 | .43 | .75 | .41 | - | .26 | .29 |
| CupB2 | .34 | .28 | .38 | .47 | .50 | .42 | .38 | .40 | .48 | .43 | - | .29 |
| SafB | .46 | .29 | .52 | .39 | .44 | .41 | .38 | .46 | .36 | .48 | .43 | - |
| 3 letter designators: Cst, ETEC coli surface antigen (CS) 3, sequence unpublished from M424C1; Cfa, ETEC colonization factor antigen (CFA) I, from NTP513/E7473; Caf1, *Y*. *pestis* capsule protein, Accession No. CAA43967.1; Hif, *H*. *influenzae* pilus, Accession No. AAA61815.1; F17a, *E. coli* fimbria F17a, Accession No. AAC45720.1 ; Pap, P pilus UPEC, Accession No. SwissProt P15319; Fas, 987P pilus, Accession No. AAB02685.1; Fim, *E*. *coli* type 1 pilus, Accession No. L14598.1; Saf, Salmonella atypical fimbriae, Accession No. CAC44269; Fae, *E. coli* K88 fimbriae, Accession No. CAA39478; Sfa, *E. coli* [UPEC] S-fimbriae, Accession No. CAC16952; CupB, *P. aeruginosa* CupB pili, Accession No. EHS38327. | | | | | | | | | | | | |
