# Supplementary material for: Structure of CfaA Suggests a New Family of Chaperones Essential for Assembly of Class 5 Fimbriae
Source: PLoS Pathog. 2014 Aug 14;10(8):e1004316. doi: 10.1371/journal.ppat.1004316 (PMC4133393; doi:10.1371/journal.ppat.1004316)
Supplement: Table S4 — Primers used in the work. (DOC) [file ppat.1004316.s005.doc]

**Table S4. Primers used in the work.**

| **Number** | **Primer name** | **Direction** | **Sequence** |
| --- | --- | --- | --- |
| 1 | cfaBmature-NdeI | Forward | 5’CCATATGCATCATCATCATCATCATGTAGAGAAAAATATT3’ |
| 1 | cfaBmature-XhoI | Reverse | 5'GCTCGAGTCAGTGATGGTGATGGTGATGGGATCCCAAAG3' |
| 2 | cfaA-NdeI | Forward | 5'GGCATATGCATAAATTATTCTA 3’ |
| 2 | cfaA-XhoI | Reverse | 5'GCTCGAGTCCAGGAACCTTTAATTG 3 |
| 3 | cfaAmature-NcoI | Forward | 5'GGCCATGGCAAACTTTATGATATATCC 3' |
| 3 | cfaAmature-NotI | Reverse | 5’GGGCGGCCGCTCAAGGAACCTTTAATTG3’ |
| 4 | CfaA/K9s | Forward | 5' AATGGAAATAGCGAGTTAGTTCGT 3' |
| 4 | CfaA/K9a | Reverse | 5' CTTTAAATCTGCTGATATTGGATA 3' |
| 5 | CfaA/T44-E46s | Forward | 5' GCAGCAGGCGTTAAAGATATTTATTTTTGTAAGTC 3' |
| 5 | CfaA/T44-E46a | Reverse | 5' AACATTCCCATTATTATAAATAATTATATTTTTTTTTGC 3' |
| 6 | CfaA/E86s | Forward | 5' AGAGTATATTTTGAGGCGGTAAAA 3' |
| 6 | CfaA/E86a | Reverse | 5' GTAAACTTCCGCTTTTTTTGGTAT 3' |
| 7 | CfaA/T112s | Forward | 5' GTTAATATAATTTATGCGGCTCTA 3' |
| 7 | CfaA/T112a | Reverse | 5' AGATAGCTCTGCTGTTAGTTTTTT 3' |
| 8 | CfaA/L114s | Forward | 5' CTAACAACAGAGGCATCTGTTAAT 3' |
| 8 | CfaA/L114a | Reverse | 5' TTTTTTATTATCAATTACATTTTC 3' |
| 9 | CfaA/V116s | Forward | 5' CTAACAACAGAGGCATCTGTTAAT 3' |
| 9 | CfaA/V116a | Reverse | 5' ATAAATTATATTAGCAGATAGCTCTGT 3' |
| 10 | CfaA/I118s | Forward | 5' CTAACAACAGAGGCATCTGTTAAT 3' |
| 10 | CfaA/I118a | Reverse | 5' ATAAATTGCATTAACAGATAGCTCTGT 3' |
| 11 | CfaA/Y120s | Forward | 5' CTAACAACAGAGGCATCTGTTAAT 3' |
| 11 | CfaA/Y120a | Reverse | 5' AGCAATTATATTAACAGATAGCTCTGT 3' |
| 12 | CfaA/R125s | Forward | 5' GCTCTAATTGCATCTTTACCAAGT 3' |
| 12 | CfaA/R125a | Reverse | 5' CGCATAAATTATATTAACAGATAG 3' |
| 13 | CfaA/R154s | Forward | 5' GATATTTATTTTTGTAAGTCATCT 3' |
| 13 | CfaA/R154a | Reverse | 5' TTTAACGCCTGCTGCAACATTCCC 3' |
| 14 | CfaA/K164-N171s | Forward | 5' GCCGCTGCTGCCTGTGTAAAAAAAGCGTAT 3' |
| 14 | CfaA/K164-N171a | Reverse | 5’AGCAGCTGCCGCACAAAAATAAATATCTTT3’ |
| 15 | CfaA/C163C172s | Forward | 5' ATCGATGATAACTCAGTAAAAAAAGCGTAT 3' |
| 15 | CfaA/C163C172a | Reverse | 5' ATTAGATGACTTTGAAAAATAAATATCTTT 3' |
| 16 | CfaA/deletion | Forward | 5’ CTAAGTTTACTCATGGCTCCCCATGAAGGCATAGAAAAAGAGC AAGGGC 3' |
| 16 | CfaA/deletion | Reverse | 5' GCCCTTGCTCTTTTTCTATGCCTTCATGGGGAGCCATGAGTAA ACTTAG 3' |
